# Tau-Marin Mucoadhesive Gel for Prevention and Treatment of Gum Diseases

**DOI:** 10.3390/gels9080607

**Published:** 2023-07-27

**Authors:** Giuseppe Giannini, Irene Ragusa, Giulia Nerina Nardone, Sara Soldi, Marina Elli, Piera Valenti, Luigi Rosa

**Affiliations:** 1 R&D Alfasigma S.p.A., Via Pontina, Km 30.400, 00071 Pomezia, Italy; 2 Labomar S.p.a., Via F. Filzi, 33, 31036 Treviso, Italy; irene.ragusa@labomar.com (I.R.); giulianerina.nardone@labomar.com (G.N.N.); 3 AAT—Advanced Analytical Technologies Srl, Via P. Majavacca 12, 29017 Fiorenzuola d’Arda, Italy; sara.soldi@aat-taa.eu (S.S.); marina.elli@aat-taa.eu (M.E.); 4 Department of Public Health and Infectious Diseases, University of Rome La Sapienza, Piazzale A. Moro 5, 00185 Rome, Italy; piera.valenti@uniroma1.it (P.V.); luigi.rosa@uniroma1.it (L.R.)

**Keywords:** Tau-Marin, mucoadhesive gel, oral formulation, probiotics, periodontitis, periodontal diseases, slow delivery

## Abstract

An innovative and stable probiotic-containing mucoadhesive gel (AL0020), integrated with botanical extracts, has been developed to rebalance the dysbiosis associated with periodontal diseases. Tau-Marin gel, prepared with anhydrous ingredients to prevent the replication of bacteria and ensure good stability over time, was tested against some pathogenic bacteria, belonging to the so-called “red complex”, recognized as the most important pathogens in plaque specimens, adherent to the epithelial lining of periodontal pockets. This lipogel was tested in vitro, in a physiological solution (PS) and in a simulated saliva (SS), for up to 8 h, to monitor its ability to release probiotics over time. Probiotics were enumerated through two different techniques, Lacto-Counter Assay (LCA) and Colony Forming Unit (CFU). A detailed physico-chemical profile of AL0020 and its in vitro efficacy in protecting activity against pathogenic bacteria as well as soothing or irritative effect on gingival epithelium were reported. Moreover, a clinical-dermatological trial on 20 volunteers using the product once a day for 30 days was also performed, where the efficacy of the gel in the control of gum disorders was observed.

## 1. Introduction

Oral diseases have remained the most dominant morbid condition globally since 1990, and among these, untreated caries of permanent teeth is the most prevalent with about 2 billion cases, followed by severe periodontal disease affecting about 1 billion cases and then untreated caries of deciduous teeth with about 510 million cases and edentulism with 350 million cases (only in 2019). The estimated combined number of oral disease cases globally is about 1 billion higher than the cases of all five major noncommunicable diseases combined (mental disorders, cardiovascular disease, diabetes mellitus, chronic respiratory diseases, and cancers). These are the data from the WHO Report on the State of World Oral Health: towards universal coverage for oral health by 2030 [1].

In such a scenario, a greater effort to find efficient and inexpensive solutions can no longer be derogated from. An alteration of the oral microbial flora (dysbiosis) plays an important role in generating health problems, both locally (caries, gingivitis, and periodontitis) and at a systemic level. Gingivitis is a condition often characterized by bleeding gums swollen and painful. If left untreated, gingivitis progresses to periodontitis which involves the loss of the periodontal ligament attachment and of the bone surrounding and supporting the teeth. Periodontal diseases are one of the most common diseases in humans, estimated to affect about 3.5 billion people [2]. These pathologies are closely associated with lifestyle, especially poor oral hygiene, medication, and alcohol consumption. [3].

A severe periodontal disease condition, with rapid loss of tooth attachment (>2 mm/year), is estimated to affect in Europe from 5 to 20% of middle-aged adults (35–44 years) and up to 40% of older people (65–74 years) [4].

Periodontal disease is one of the major causes of tooth loss with important effects on mastication, aesthetics, self-confidence, and quality of life. In the past decade, evidence of a connection between periodontal disease and oral biofilm, prodromal a periodontal disease, and those on the systemic conditions, either initiating or complicating this pathology, are increased. These multiple health conditions include cardiovascular and renal issues, diabetes, osteoporosis, pulmonary disorders, Alzheimer’s disease, adverse pregnancy outcomes, rheumatoid arthritis, chronic obstructive pulmonary disease, preeclampsia in pregnant women and pre-term pregnancy, prostate cancer, colon cancer, pancreatic cancer, erectile dysfunction, and other systemic conditions [5,6,7].

Although periodontal disease can be prevented, patients pay attention to it and turn to dentists only when the infection is in an advanced stage, with the appearance of lowering of the gingival collar, hypersensitivity, bleeding, pain, and mobility of the teeth [8].

More than 80% of the risk of periodontal tissue damage is due to an altered or inadequate response of the oral microbiota to the attack of pathogens, promoters of periodontitis. Various pathogens are involved in periodontal disease. These pathogens mostly live in biofilm in human mouth thus aggravating their pathogenic activity.

The so-called “red complex” [9] has been proposed as a pathogenic consortium, consisting mainly in anaerobic facultative intracellular pathogens as *Porphyromonas gingivalis*, *Aggregatibacter actinomycetemcomitans*, *Treponema denticola*, *Tannerella forsythia*, and *Prevotella melaninogenica*, bacteria that have the ability to survive and grow intracellularly in the host cells, thus causing tissue damage and escaping the host’s natural defense mechanisms. They preferentially colonize the subgingival sites in the oral cavity [10].

Live probiotics could play a very important role in the oral cavity in prophylaxis and therapeutic use to treat a variety of diseases. As matter of fact, probiotic bacteria impact on human health, so that their application on preventing or treating oral diseases appears to be a very promising new therapy approach [11]. Various oral dispersion probiotics have been proposed in recent years with the aim of enriching the oral microbiota with “good” bacteria (commensals) in order to counteract “bad” bacteria (pathogens). In fact, periodontitis is mostly related to disproportionate microflora resulting in overgrowth of periodontal pathogens belonging to the “red complex” [12].

In addition, the recent literature has documented the potential of antioxidants in highbush blueberries’ proanthocyanidins (PACs), which have proven to be effective against bacteria associated with an aggressive form of gum (periodontal) disease and biofilm formation. Antioxidants from blueberries were shown to protect oral macrophages which are crucial to the immune system. The PACs also blocked the molecular pathway involved in inflammation, that is in turn largely involved in gingival and periodontal disease [13]. From the results, the authors hypothesized the oral use of PACs or blueberry extracts in reducing inflammation and bacterial growth.

An equally interesting profile has been described for aloe extracts, characterized by protective and anti-inflammatory properties. Several clinical studies have demonstrated their effectiveness in the prevention and treatment of gingivitis and periodontitis by reducing gingival index, plaque index, and probing depth and by increasing bone fill and regeneration [14,15,16,17]. A randomized clinical trial has been recently reported where an oral gel, loaded with postbiotics, lactoferrin, and aloe barbadensis leaf extract, showed an efficacy equivalent to a comparative chlorhexidine-based gel for the domiciliary treatment of periodontitis [18].

The main objective of an oral dispersion of probiotics (eubiosis) is to counteract the pathogenic bacteria responsible for oral dysbiosis. For this purpose, we considered that a prolonged probiotics dispersion was much more efficient than the dispersion lasting few minutes.

The results presented here are the continuation of a previous exploratory study, where it was demonstrated that probiotics were stable over time (more than one year) and were slowly released into the oral cavity after application, if formulated in an anhydrous mucoadhesive gel. We had also demonstrated the effectiveness of this formula in contrasting bone reduction following an inflammatory stress and in counteracting—in vitro—some pathogenic bacteria belonging to the “red complex” [19].

Here, we report the results obtained with a new formula (lab code: AL0020) containing probiotics and botanical extracts, where the mucoadhesive gel has been loaded with three probiotics at 1%, *Lacticaseibacillus rhamnosus* SP1 (*L. rhamnosus* SP1), *Lactobacillus helveticus* SP27 *(L. helveticus* SP27), and *Lacticaseibacillus paracasei* CBA-L87 *(L. paracasei* CBA-L87), so defined according to the latest nomenclature [20], and with three botanical extracts, Aloe Barbadensis leaf, Vaccinium Myrtillus Fruit, and Malva Sylvestris Leaf. The final formula (AL0020) was evaluated both in in vitro tests (competition, mucosa, and epithelia) and in a survey of 20 volunteers who used Tau-Marin gel (AL0020) once a day for 30 days, with a positive response based on the protective effect, a reduction in gingival inflammation and bleeding, associated with a better general condition of gum health. Also, the gel was appreciated without any revulsion.

## 2. Results

### 2.1. Mucoadhesive Gel (AL0038) and Ingredients

The mucoadhesive gel (AL0038) does not contain any active ingredients but consists of an oily basic gel inside of which mucoadhesive polymers are dispersed. The resulting gel is viscous and beige/ivory in color and menthol flavor. The composition of mucoadhesive gel (AL0038) is reported in Table 1.

### 2.2. Tau-Marin *Mucoadhesive Gel* (AL0019, AL0039, and AL0020) Preparation

To the previous basic formulation (AL0038) were added three botanical extracts in powder (Aloe vera leaf extract, blueberry extract, and mallow leaf extract). The resulting lipogel is AL0019, viscous and beige/ivory in color, with green and violet dots and menthol flavor.

At the same time, the basic formulation (AL0038) was added with three probiotics (*L. rhamnosus* SP1, *L. helveticus* SP27, and *L. paracasei* CBA-L87). The resulting product is AL0039, viscous and beige/ivory in color and menthol flavor (Table 2 and Table 3).

At the end, to the basic formulation (AL0038) were added both three botanical extracts and three probiotics. The resulting final formulation of lipogel is AL0020, viscous and beige/ivory in color, with green and violet dots and menthol flavor.

### 2.3. Antimicrobial Activity: Antagonist Effects

In Table 4, a scheme of each combination evaluated in the competition test was reported. Chlorhexidine digluconate 0.2% was included as positive inhibition control.

Results obtained from the competition tests (inhibition zone) were consistent with those obtained in the previous project [19], confirming the anti-bacterial activity of the tested ingredients of the product, although evaluated by means of in vitro assays. In the second assessment, the antimicrobial action of the single herbal extracts, of the probiotic mixture, and of the different matrices was measured.

The results of the agar diffusion well test are reported in Table 5, showing the different inhibitory effect of the gels compared to the positive control, chlorhexidine (CHX).

As shown in Table 5, a neutral gel (AL0038) and the two gels containing the separate probiotic mixture (AL0039) or botanical extracts (AL0019) showed a reduced inhibiting power compared to the complete formulation AL0020 against the single components of the “red complex” as well as against the pathogen blend.

An inhibition test provided interesting results in order to understand the contribution given by the individual components in contrasting the pathogenic bacteria of the oral cavity. The probiotic strains, *L. rhamnosus*, *L. helveticus*, and *L. paracasei*, individually tested showed minimal inhibition on some representatives of the “red complex”. The efficacy was increased towards single pathogenic strains examined when the same three probiotics were added into the mucoadhesive gel (AL0039), also including an efficacy on the pathogenic bacteria mixture (blend). Similar results were observed by the single botanical extracts, and again the efficacy was extended to all pathogens when single extracts were mixed in the same matrix (AL0019).

In detail, Aloe vera and mallow leaves powders weakly inhibited all pathogens and their blend, with the only exception of *T. denticola*. Blueberry extract weakly inhibited the pathogens’ blend and *P. melaninogenica*, without any influence on the other members of the “red complex”.

The pathogens’ blend clearly showed that the so–called “red complex” was actually able to create a very efficient biofilm, in which the different strains were integrated and able to optimize growth even in less favorable conditions, as the presence of potentially inhibiting agents.

However, the pathogens’ blend was demonstrated to be more prone to inhibition when all the ingredients (probiotics and botanicals) were added into the mucoadhesive gel: in fact, the final formulation (AL0020) turned out to be the best performing, and its action/efficacy was extended to the *A. actinomycetemcomitans*.

These results support the hypothesis that Tau-Marin gel (AL0020) could be a very effective device in counteracting the growth of pathogenic bacteria in the oral cavity.

### 2.4. Enumeration of Bacteria in Tau-Marin (AL0020) *Mucoadhesive Gel* by LCA and CFU

As described above, probiotics were enumerated in Tau-Marin gel (AL0020; batch L1538 K) by means of two different techniques, CFU and LCA, as shown in Table 6 and Table 7.

These analyses were repeated over time, in order to monitor the stability of the product: as previously mentioned, in a recent work we demonstrated that bacteria were stable within the gel for 12 months, while the evolution of the stability of this new formulation is currently ongoing, scheduled until the 36th month. New stability data related to the first twelve months (T12) are displayed in Table 6 and Table 7, evaluated as bacteria release in PS or SS, respectively.

Furthermore, as additional information on the stability left for 18 months at room temperature using an equivalent formulation (AL0017), previously prepared without adding Aloe vera, the bacterial load was reduced only slightly, confirming a high stability of this formulation.

In particular, the composition of AL0017 (batch IR0192B) is AL0038 + *L. rhamnosus* SP1 (1%), *L. helveticus* SP27 (1%), *L. paracasei* (1%), mallow leaves (0.6%), and blueberries (0.4%), and the stability is 3.1 ± 0.2 × 10^9^ at T18 compared to 4.1 ± 0.1 × 10^9^ at T0.

### 2.5. In Vitro Evaluation of Irritative Potential, Protective Efficacy, and Soothing Effect on Gingival Epithelium

#### 2.5.1. Inserts of Gingival Epithelium Reconstructed In Vitro

To evaluate the irritating/protective effect of gel on an in vitro gingival model, an in vitro reconstructed epithelium of human gingiva with a surface area of 0.50 cm^2^ was used, placed on a support consisting of a cellular multilayer. All were placed on top of an inert polycarbonate filter. This cellular multilayer histologically simulates the superficial part of the human gingiva.

In the present study, technical data of the Skinethic^®^ HGE gingival epithelium were used.

The inserts of gingival epithelium reconstructed in vitro were treated topically with the sample under analysis for 4 h. At the same time, analogous inserts were treated with physiological solution for 1 h, as negative control, and with Sodium dodecyl sulphate solution (SDS 0.5%) for 1 h, as positive irritative control. At the end of each kind of exposure, the sample or the control solutions were removed from the surface of the inserts with a wash in saline, followed by gentle drying.

At the end of the 24 h rest period after treatment, cellular viability has been checked in all inserts analyzed using MTT titration, and the cellular medium was collected and analyzed for IL-1α expression.

Tau-Marin gel (AL0020) did not lead to a reduction in cell viability below 50%, which is placed as a criterion of acceptability for positive control. It can therefore be considered that the treatment with sample AL0020 did not affect the cell viability (Table 8).

In addition, the treatment of gingival epithelium inserts with the irritative agent, consisting of a 0.5% aqueous solution of SDS, led to a drastic reduction in cellular viability of the multilayer. The pre-treatment of the inserts with Tau-Marin gel, followed by the application of the irritant, always consisting of the aqueous solution of 0.5% SDS, did not lead instead to a reduction in cell viability, compared to the negative control used as a reference. Therefore, the pre-treatment with the test sample determines an increase compared to the treatment performed only with the irritant, using the same time of application of the latter (Table 9).

#### 2.5.2. Human IL-1 Alpha ELISA Test

The gel AL0020 was applied on the surface of the gingival epithelial layer reconstructed in vitro. The objective of the test was to evaluate the ability of gel to penetrate the cellular multilayer, affecting the living cells of the deeper layers. By applying the gel after having subjected the cell layer to an irritation treatment, it is possible to evaluate whether it has a soothing action. If, on the other hand, the gel is applied before the irritant treatment, it can be assessed whether it can form a protective barrier.

Evaluation of irritative potential of the sample: The inserts of gingival epithelium reconstructed in vitro were treated topically with the sample under analysis for 4 h. At the same time, analogous inserts were treated with physiological solution for 1 h, as a negative control, and with sodium dodecyl sulphate solution (SDS 0.5%) for 1 h, as a positive irritative control. At the end of each kind of exposure, the sample or the control solutions were removed from the surface of the inserts with a wash in saline, followed by gentle drying.

At the end of the 24 h rest period after treatment, cellular viability was checked in all inserts analyzed using MTT titration.

Evaluation of protective efficacy of the sample: The inserts of gingival epithelium reconstructed in vitro were treated topically with the sample under analysis, in sufficient quantity to cover the surface, followed by sodium dodecyl sulphate solution (SDS 0.5%) for 1 h. At the same time, analogous inserts were treated with physiological solution for 1 h, as a negative control, and with SDS 0.5% solution for 1 h, as a positive irritative control. At the end of each kind of exposure, the sample or the control solutions were removed from the surface of the inserts with a wash in saline, followed by gentle drying.

**Figure 1:**
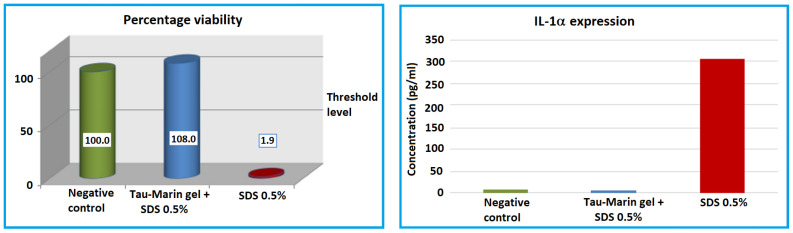
Viability and IL-1a expression of Tau-Marin gel versus negative and positive controls on a gingival epithelium model, used as protective agent against an irritative agent (0.5% SDS).

At the end of the 24 h rest period after treatment, cellular viability was checked in all inserts analyzed using MTT titration, and the cellular medium was collected and analyzed for IL-1α expression (Figure 1).

The gingival epithelium, that undergoes treatment with the irritative agent (0.5% SDS), after treatment with Tau-Marin gel maintains a cellular viability and an IL-1αexpression similar to that evidenced by the inserts used as negative control, whereas the inserts treated only with the irritative agent show a significant decrease of cell viability and increase of IL-1α levels.

Evaluation of soothing effect of the sample: Gingival epithelium inserts underwent a pre-treatment with an irritant factor (lactic acid 1.0% for two hours) and, subsequently, were washed with saline and dried gently.

To verify the soothing properties of the sample, two tissue inserts were treated with the sample analyzed, in sufficient quantity to cover the surface. The sample was applied to the epithelial inserts, and the exposure was maintained for four hours and was repeated for two days. At the end of each period of exposure to the sample, it was removed from the surface of the insert with a wash in saline, followed by gentle drying.

At the same time, two inserts were exposed to a treatment with acetylsalicylic acid (0.03% solution) for four hours as reference sample for a soothing effect.

**Figure 2:**
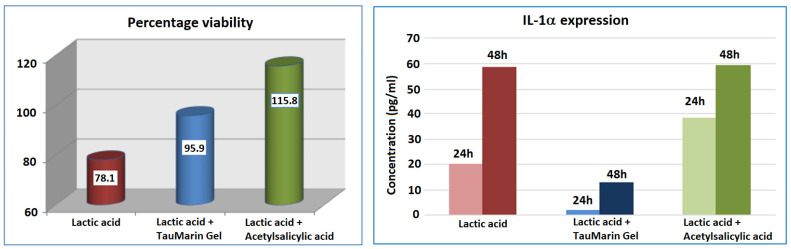
Viability and IL-1a expression of Tau-Marin gel versus a negative and positive controls on a gingival epithelium model, used as a soothing agent in counteracting the effects of an irritant agent (0.5% SDS).

At the end of the 48 h rest period after the initial irritative treatment, cellular viability was checked in all inserts using MTT titration, whereas the cellular medium was collected and analyzed for IL-1 expression at 24 and 48 h after the initial treatment (Figure 2).

The gingival epithelium that undergoes treatment with the irritative agent (1.0% Lactic acid) demonstrates reduced cell viability and increased IL-1α expression. The treatment with Tau-Marin Gel, after the irritative agent, shows a rise in cellular viability and the lowering of IL-1α expression. The percentage viability achieved and the low expression of IL-1α allows for the attribution of an effective barrier action in the sample.

### 2.6. In Vitro Evaluation of Irritation on Human Oral Mucosa and TEWL Measurement

This test is based on in vitro reconstructed oral mucosa inserts. These are inserts with a surface area equal to 0.50 cm^2^ of mucosa reconstituted in vitro from transformed human keratinocytes from a squamous cell carcinoma of the buccal mucosa.

This support consists of a cellular multilayer developed on top of an inert polycarbonate filter. The cellular multilayer histologically resembles the mucous membrane present in the oral cavity and, like this one, is devoid of a stratum corneum. It is therefore possible to reproduce in vitro the effect that the application of a product could have in vivo on the oral mucosa.

The MTT test measures the amount of formazan formed in cell culture. And this provides a measure of cell viability. For the MTT test to be valid, the following acceptance criteria for controls must be met:

- Negative control:

The average optical density of the extracted solution at the end of the MTT test must be greater than 0.8 and less than 3.0. The standard deviation should not exceed 18%.

- Positive control:

The percentage viability, obtained by comparing the optical density of the extracted solution at the end of the MTT test with that relating to the negative control, must be less than 50%, and the standard deviation of the percentage viability must not exceed 18%.

**Figure 3:**
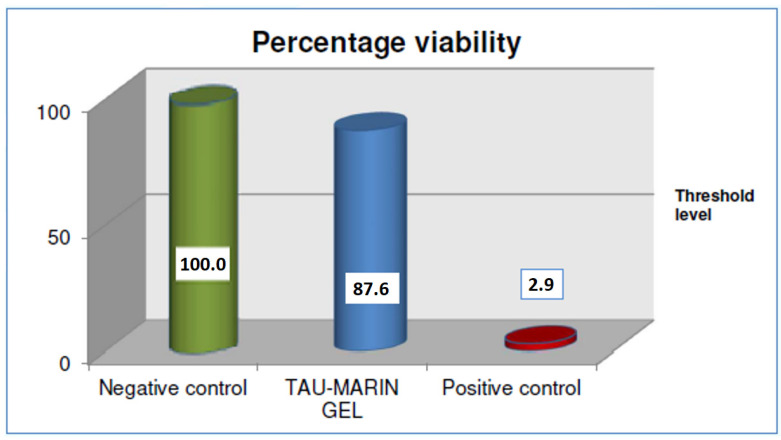
Irritative effect of Tau-Marin gel versus negative and positive control, evaluated on a human oral mucosa model after an application lasting four hours.

The sample under analysis (Tau-Marin gel) is considered not irritating on oral mucosa with viability results of 87.6% after an application lasting four hours, followed by the post-treatment rest period as it shows, compared to the negative reference control (Figure 3).

TEWL (Transepidermal water loss), measured by the Vapometer-Delfin, is a key indicator of skin barrier function, and the ability to measure it accurately is essential in a wide range of clinical and personal care applications. In this test, the measurement of TEWL was performed on the mucosal inserts to verify their greater or lesser surface permeability.

For the TEWL test to be valid, its value for the positive control inserts must be higher than the value for the negative control inserts after treatment.

The trend of TEWL in the inserts treated with the sample shows a substantial maintenance of the barrier function of the treated mucosa throughout the study time (Table 10 and Figure S3 in S.I.).

The TEWL values remain optimal and comparable to those of the negative control, indicating a non-alteration of the barrier function of the mucous membrane, both at the end of the four hours of application and after the post-treatment rest period.

### 2.7. Clinical Evaluation, Assessment of the Microbiome, and Self-Assessment Questionnaire of a Cosmetic Product for Oral Use

#### 2.7.1. Selection of Volunteers

Recruitment and admission criteria: tests were performed in accordance with the Declaration of Helsinki on 20 volunteers, with an average age of 43.5 years. Subjects were informed about nature, purpose, and risks of the study. They were required to give their written consent before participating in the test.

The selection of subjects was carried out in accordance with the following criteria:

(a) Inclusion criteria
  - Caucasian subjects;
  - Males and females between 18 and 70 years of age, in good general health;
  - Subjects able to follow all the instructions of the study and to commit to carry out the scheduled visits for the entire duration of the study;
  - Subjects who give their informed consent (Study N° RAP23816);
  - Subjects with alteration of the gingival mucosa (e.g., erythema, erosions, leukoplastic lesions, and bleeding);
  - Subjects with or without tartar, dental interventions, prostheses, etc.
(b) Exclusion criteria
  - Pregnant or nursing women;
  - Subjects with a history of particular skin reactions to cosmetic products and detergents or with sensitivity to one of the components of the product;
  - Subjects who are taking topical or systemic medicines that may interfere with the results of the tests (anti-inflammatory agents, cortisones, antibiotics, etc.);
  - Subjects who show systemic diseases or skin disorders (eczema, psoriasis, dermatitis, etc.);
  - Subjects who currently use adjuvant treatments for the well-being of gums or who have used them in the last three months before the start of this study (neither topical nor systemic);
  - Diabetic subjects;
  - Smokers and habitual consumers of alcoholic beverages;
  - Subjects who have participated in other similar studies in the period of 30 days prior to this.
(c) Drop out—reasons considered sufficient to terminate the participation of the subjects in the study:
  - Free choice of subject;
  - Medical reasons unrelated to treatment (e.g., onset of disease or surgery);
  - Reasons related to treatment (e.g., irritation or allergic reactions).

Restrictions: During the study, the subjects are instructed not to use hygiene products of the teeth and of the entire oral cavity (e.g., mouthwashes, pastes, chewing gum with xylitol, etc.) other than those delivered and not to apply the products under examination in parts other than those prescribed.

#### 2.7.2. Instruments and Parameters

a. Clinical-Dermatological evaluation.

At the two evaluation times (start and end of treatment) a clinical-dermatological evaluation was carried out on four parameters in the oral cavity: (a) erythema, (b) erosions, (c) leukoplastic lesions, and (d) gum bleeding. The dermatological evaluation is expressed according to the following scale of values: 0 = absent; 1 = mild; 2 = moderate; 3 = severe.

The effectiveness of the product in improving the well-being of the oral cavity is evidenced by the decrease in the values of the parameters at the end of the treatment for erythema and gum bleeding, whereas it is supported by the maintenance of absence of erosions and leukoplastic lesions.

b. COPAN Swabs sampling and transport system.

The COPAN Swabs sampling and transport system is intended for the collection, transport and storage of clinical samples that must be analyzed with nucleic acid amplification techniques. Sampling was performed on the upper and lower gums.

c. Subjective evaluation.

At the end of the test, the volunteers expressed their subjective opinion on the effectiveness and pleasantness of the treatment by filling out a self-assessment questionnaire. For each question, the volunteers expressed their opinion on a four-point rating scale corresponding to four different intensities perceived according to the following values: 1 = insufficient; 2 = sufficient; 3 = good; 4 = excellent.

#### 2.7.3. Method

a. Study design.

Twenty subjects selected as referred to in paragraph 3 were instructed on the use of the product (Tau-Marin gel) as follows: application of gel in the evening, before bedtime, and after brushing your teeth with the neutral toothpaste. Use your finger to apply the gel on the collar, between the tooth and gum. Wait a few seconds for a protective film to form, humming with saliva. After the night’s rest (about 8 h), when you wake up, remove the gel with your toothbrush.

b. Evaluation method

b.1 Clinical-dermatological evaluation.

All evaluations are performed at the beginning (T0) and at the end of the treatment (TF).

Clinical-dermatological evaluation of the four parameters is performed on the oral cavity of the subjects at T0 and TF control times and is expressed through an evaluation scale. The collected numerical parameters are statistically evaluated. The rebalancing action of the products is evidenced by the improvement compared to the initial conditions of the clinical-dermatological evaluation data regarding erythema and gum bleeding and the maintenance of low values of leukoplastic lesions and erosion evaluations.

b.2 COPAN Swabs sampling.

Copan Swabs are performed at home by each subject at T0 and TF following the instructions of the supplier company. The area of the oral cavity involved in the test is the upper and lower gum area. Protocol for sampling with Copan Swabs: Refrain from any oral hygiene maneuver in the 24 h prior to the swab. Perform the last application of Tau-Marin gel 36 h before the swab. Swabs stored at 4 °C are delivered to the laboratory for the detection of nucleic acids.

b.2.1 DNA e × traction.

DNA was extracted using Qiacube HT robot and Cador Pathogen 96 QIAcube HT Kit with a modified lysis step. Some 300 μL of the eNAT preservative buffer (Copan ITA) was added with 100 μL of zirconia beads and 750 μL of Bead Solution and 60 μL of C1 solution (PowerFecalR (Qiagen)). Samples were incubated at 65 °C for 10 min and shaken for 10 min on the Tissue Lyser (Qiagen) at 25 Hz. Finally, samples were centrifuged at 13,000× *g* for 1 min, and 200 μL of the supernatant was used as starting material for the extraction, following manufacturer instructions.

b.2.2 DNA amplification.

Some 5 μL of eluted DNA was used for the amplification. The V3-V4 regions of the 16S ribosomal RNA gene were amplified using Illumina tailed primers

Pro341F (5′-TCGTCGGCAGCGTCAGATGTGTATAAGAGACAG-CCTACGGGAGGCAGCA-3′) and Pro805R (5′-GTCTCGTGGGCTCGGAGATGTGTATAAGAGACAGGACTACNVGGGTATCTAATCC-3′) using HiFi Platinum Taq (Thermo Fisher Scientific Inc., Waltham, MA, USA) via PCR (94 °C for 2 min, followed by 25 cycles at 94 °C for 30 s, 55 °C for 30 s, and 68 °C for 30 s and a final extension at 68 °C for 7 min) [21].

b.2.3 Library Preparation and Sequencing.

PCR amplicons were purified with Magnetic Beads Agencourt XP (Beckman Coulter, Inc., Brea, CA, USA), diluted 1:2 and amplified following the Nextera XT Index protocol (Illumina, Inc., San Diego, CA, USA). The amplicons were normalized by the SequalPrep™ Normalization Plate Kit (Thermo Fisher Scientific Inc., Waltham, MA, USA) and multiplexed.

The pool was purified with 1X Magnetic Beads Agencourt XP (Beckman Coulter, Inc., Brea, CA, USA), loaded on the MiSeq System (Illumina, Inc., San Diego, CA, USA), and sequenced following the V3-300PE strategy.

b.3 Reading the questionnaire.

Data collected are represented graphically as absolute frequencies of express judgment and are shown in the table in form of relative percentage frequency (Table 11).

#### 2.7.4. Results

Regarding clinical evaluation, assessment of the microbiome, and self-assessment questionnaire of a cosmetic product for oral use, in order to simplify the interpretation of the results, a rating scale is used for each parameter with the following values: 1 = Insufficient, 2 = Sufficient, 3 = Good, 4 = Excellent.

c. Mathematical elaboration.

c.1 Clinical-dermatological evaluation.

The data obtained were statistically processed using the statistical program R-studio. For significance analyses, the Wilcoxon signed rank test was applied for paired data with a significance level of *p*-value < 0.05.

All the parameters considered were assigned a rating score according to the following four-point scale: 0 = absent; 1 = mild; 2 = moderate; 3 = severe.

After 30 days of treatment with the product, since *p*-value < 0.0001, it can be concluded that there is a significant change in the average evaluation rating of the presence of erythema, and therefore the hypothesis is accepted that data relating to this value after 30 days are different from those before use.

The average decrease in the assessment of the presence of erythema between T0 (initial) and TF (after 30 days of treatment) corresponds to −0.8 (Table 12).

A total of 100% of the volunteer subjects showed a significant improvement in the erythema evaluation (decrease) after 30 days of treatment.

After 30 days of treatment with the product, since *p*-value = 0.0003, it can be concluded that there is a significant change in the average evaluation rating of the presence of gum bleeding, and therefore the hypothesis is accepted that data relating to this value after 30 days are different from those before use.

The average decrease in the assessment of the presence of gum bleeding between T0 (initial) and TF (after 30 days of treatment) corresponds to −1.2 (Table 13).

A total of 90% of the volunteer subjects showed a significant improvement in the gum bleeding evaluation (decrease) after 30 days of treatment.

After 30 days of treatment with the products, since *p*-value = 0.35, it can be concluded that there is no significant change in the average value of the presence of erosions, and therefore the hypothesis is accepted that data relating to this value after 30 days are equal to those before use.

Treatment is proven not to cause erosion during its use (Table 14).

After 30 days of treatment with the products, since *p*-value = 0.37, it can be concluded that there is no significant change in the average value of the presence of leukoplastic lesions, and therefore the hypothesis is accepted that data relating to this value after 30 days are equal to those before use. Treatment is proven not to cause leukoplastic lesions during its use. A total of 100% of the volunteer subjects showed that they maintain or improve their rating in the presence of leukoplastic lesion evaluation after 30 days of treatment (Table 15).

c.2 Bioinformatics analysis after DNA amplification.

The whole data analysis workflow was performed using QIIME2 v2021.4. Raw reads were processed with cutadapt in order to remove primer sequences and were subsequently filtered, denoised, merged, and cleaned by chimera with *DADA2*, run with default parameters (--p-trunc-len-f 270, --p-trunc-len-r 215). The obtained amplicon sequence variants (ASVs) were then filtered by frequency applying a 0.01% threshold to remove singletons and poorly represented sequences. The feature-classifier plugin was applied to assign the proper taxonomy to ASVs, using trained OTUs at 99% from Silva (v 138) and Green Genes (v 13-8) databases. After the selection of the most feasible value, the feature table (sample in columns and ASV in rows) was rarefied, and alpha diversity indices (observed features, evenness, Faith PD, and Shannon) and beta diversity metrics (weighted and unweighted unifrac, Bray–Curtis, and Jaccard) were calculated using the diversity plugin. Moreover, statistical comparisons among the two time points were assessed with Kruskall–Wallis and Permanova tests, for alpha and beta diversity, respectively, while pairwise differences were calculated using the Wilcoxon signedrank test, integrated in the longitudinal plugin. Beta diversity PCoAs were calculated using the upgma clustering method with 200 iterations and drawn with the EMPeror visualization tool. Finally, differential abundance analysis was performed with ANCOM, collapsing the feature table at various levels (features, species, genus, family, and phylum).

d. Drop out cases.

One subject stopped the treatment for personal reasons that arose after the start of the test but not related to the products used. Therefore, it has not been possible to collect the subjective evaluation in the self-assessment questionnaire from this subject. Moreover, the data relating to the measurements of this subject, initially collected, were included in microbiome evaluations at initial time (T0) but were not included in the statistical evaluation comparing date of initial time (T0) with those at the end of the testing time (TF). For the same reason, data relating to this subject were not included in clinical-dermatological evaluations, as they are expressed as comparative data between T0 and TF.

#### 2.7.5. Microbiome Analysis

The study was performed to evaluate microbiota changes between two time points (T0 and TF). Overall, the investigations showed a stability of the microbial composition along the time, as alpha (S.I.; Figure S1A,B) and beta diversity (S.I.; Figure S1C) analyses and taxa differential abundances evaluation did not highlight significant differences.

Compositional data analysis of microbiota specimens was made by using ANCOM. In this way, we assessed if some taxa, at various levels (i.e., phylum and genus) (S.I.; Figure S2), were significantly different between the two time points. Confirming previous results, no taxon was found to change its abundance significantly, even if some differences are visible analyzing grouped bar plots at family and genus levels.

In conclusion, we can affirm that the sample microbial composition has not undergone significant modifications between the two time points.

The non-variability of the microbiota following treatment can be seen as a positive result because, if the gum discomfort were not of microbiological origin, the treatment would eliminate/minimize the cause of the problem, without significantly changing the physiological composition of the microbiota. Otherwise (problem of microbiological origin), the lack of significance is not total, and therefore the reduction of gum problems in patients may partly derive from the improvement of alpha diversity indices that are significant by analyzing the samples in a coupled way.

## 3. Discussion

The results presented here are in continuation of what has already been published by our group, in a previous project aimed at the development of a mucoadhesive gel (AL0005), where we observed that probiotics are effective and counteract the action of bacterial pathogens, which had to be loaded into the lipogel at a concentration of not less than 1%. The formulation loaded with probiotics (blend) at a concentration equal to 2% and integrated with botanical extracts was stable over time. This lipogel, after application on the gingival mucosa, was able to release the probiotics slowly and constantly. This gel was found to have an antibacterial action, evaluated on some bacteria components of the plaque, the so-called “red complex”, which starts on the gingival collar, threatening the health of the mouth and the stability of the teeth.

Now, here we present the results of an improved formulation (AL0019) where the overall bacterial load has increased to 3% (three bacteria at a concentration of 1% each), and the composition of the botanical extracts has also been slightly modified with the aim of improving its plasticity (spreadability) and color. In fact, for this purpose, some natural ingredients have been replaced with respect to the previous formulation.

This new formulation (AL0020) has been tested both in different in vitro tests and in a clinical study on 20 volunteers.

The ability of probiotics, added to the gel, to compete with some of the bacteria belonging to the “red complex”, was evaluated in vitro, and, interestingly, the individual components were less efficient than the final formulation in counteracting the development of pathogens.

The stability of the formulation over time, detected by CFU and LCA, was evaluated. This formulation remains stable at room temperature at least up to twelve months. We are confident that this new formulation could be stable over 18 months similarly to that already observed with the first formulation.

The gel was also subjected to a series of in vitro tests on safety, calming effect, and irritability (TEWL). In all tests, the formulation was found to be non-aggressive but, on the contrary, endowed with a protective effect.

Finally, the AL0020 gel was tested on a group of 20 volunteers, under medical supervision, who applied the gel to their gums every evening for 30 days and, at the end of the treatment, the dentist expressed an opinion on the health of their mouth. The result, evaluated with a self-assessment questionnaire, was positive. Both the dentist and the volunteers observed that after a month there was less bleeding, as well as an apparent improvement in the health of their gums, such that they considered the gel pleasant and not unpleasant.

Furthermore, saliva samples were analyzed microbiologically, at the beginning and after 30 days, at the end of the study. The results showed a stability of the buccal microbial composition along the time, as alpha and beta diversity analyses and taxa differential abundances evaluation did not highlight significant differences. This non-variability of the microbiota following treatment can be seen as a positive result because, if the gum discomfort were not of microbiological origin, the treatment would eliminate/minimize the cause of the problem, without significantly changing the physiological composition of the microbiota. Otherwise, the lack of significance is not total, and therefore the reduction of gum problems in patients may partly derive from the improvement of alpha diversity indices that are significant by analyzing the samples in a coupled way.

## 4. Conclusions

One of the most recent approaches to the treatment of oral dysbiosis, responsible for a series of pathologies of the oral cavity, starting from gingivitis up to periodontitis, is the local administration of probiotics or botanical extracts.

Some bacteria have been identified as the main responsible for oral dysbiosis and components of the so-called “red complex”, consisting mainly of anaerobic facultative intracellular pathogens such as *Porphyromonas gingivalis*, *Aggregatibacter actinomycetemcomitans*, *Treponema denticola*, *Tannerella forsythia*, and *Prevotella melaninogenica* constituting bacterial plaque.

These are some of the bacteria described in the literature for their ability to colonize subgingival sites, penetrate inside the host cells, and thus elude the host’s defense system, causing chronic inflammation with tissue damage. Thus, periodontitis may be associated with multifactorial chronic inflammation caused by oral dysbiosis.

Local administration of exogenous bacteria (probiotics) can promote oral eubiosis. For this purpose, botanical extracts have also been described as effective.

Previously, we had already described the results of a project aimed at developing a mucoadhesive gel that would keep the bacteria loaded in it viable and that would be able to release them regularly once the gel was applied to a mucosa, obviously in addition to having identified the most effective probiotics.

The new formulation (AL0020), described in this manuscript, has a higher bacterial load (3%) in addition to botanical extracts, Aloe, blueberry, and mallow.

The formulation was found to be stable, if kept at room temperature, non-aggressive on the gingiva models where it was tested, endowed with a protective effect, and capable of reducing inflammation (IL-1).

Furthermore, it was evaluated on a group of twenty volunteers who applied the gel to the gums for 30 days, every night after brushing their teeth and before going to sleep. The result was a reduction in gingival bleeding and a better condition of gum health. Also, the gel was appreciated without any revulsion, after evaluation with a self-questionnaire.

## 5. Material and Methods

### 5.1. Mucoadhesive Gel (AL0038) Preparation

For the preparation of the mucoadhesive gels, botanical extracts were used together with cometic-grade ingredients compatible with the oral cavity. These ingredients are normally used in marketed products, such as toothpastes. To improve the stability of the lipogel, modified silicas (MPs) such as silica dimethyl silylate (AEROSIL^®^ R972) and hydroxypropyl methylcellulose (HPMC) (BENECEL K100M) were also used, as described in Table 1.

### 5.2. Tau-Marin *Mucoadhesive Gel* (AL0020) Preparation

We have previously reported [19] the preparation of a basic mucoadhesive gel, (AL0005). Now, the formula has been slightly modified, and mint has been chosen as a flavor (Lab code, AL0038).

To this basic formulation were added the three botanical extracts (Lab code, AL0019). Alternatively, to AL0038 the three probiotics (AL0039) were added. In both cases, the final formulation (AL0020) contains botanical extracts and probiotics, as indicated in Table 2. In particular, the gel was loaded with the three selected probiotics, *L. rhamnosus* SP1 (1%), *L. helveticus* SP27 (1%), and *L. paracasei* CBA-L87 (1%), as indicated in Table 2 and Table 3.

The selection criteria of the three botanical extracts were different. Aloe vera and blueberry extract are supported by the literature data for their effectiveness in promoting well-being of the oral cavity through a high effect in protecting the teeth against bacteria responsible for accelerating tooth decay, [22,23,24] while the Malva Sylvestris leaf extract was selected for its emollient, [25] anti-inflammatory, antioxidant, and osteoblast differentiation properties [26].

The final formulation was added with “menta flavour” to give a pleasant taste.

It is important to underline that this formulation is water-free in all the phases of the preparation. This feature is essential to inhibit bacterial proliferation, thus ensuring that the formulation has prolonged stability over time at room temperature.

### 5.3. Pathogenic and Probiotic Strains

Probiotic strains, namely *L. rhamnosus* SP1, *L. helveticus* SP27, and *L. paracasei* CBA-L87, key bioactive components of this mucoadhesive gel, in lyophilized form, were evaluated individually. Five pathogenic bacteria, from the “red complex” consortium, were selected for this competition test. Pathogenic strains (*Prevotella melaninogenica* DSM 7089, *Tannerella forsythia* DSM 102835, *Porphyromonas gingivalis* DSM 20709, *Treponema denticola* DSM 14222, and *Aggregatibacter actinomycetemcomitans* DSM 8324) were purchased from German Collection of Microorganisms and Cell Cultures GmbH (DSMZ, Braunschweig, Germany) and propagated in Columbia blood agar base (Oxoid, Thermo Fisher Scientific, Altrincham, UK), supplemented with 5% defibrinated horse blood to check their growth and purity in anaerobic chamber (atmosphere composed by 80% N_2_, 10% CO_2_, and 10% H_2_).

*T. forsythia* DSM 102835 required N-Acetylmuramic acid NAMA (Merck KGaA, Darmstadt, Germany) for its growth, and for this reason, for all the experiment, NAMA was added to the medium at a final concentration of 10 µg/mL. Tested items are indicated in Table 16. The agar well diffusion test on the above-described strain was performed using the same approach followed in our previous work [19].

### 5.4. Enumeration of Bacteria in Tau-Marin *Mucoadhesive Gel* by LCA and CFU

Lactobacilli, contained in the lipogel formulations (AL0020), were counted by both Lacto-Counter Assay (LCA) and Colony Forming Unit (CFU) method.

The need to evaluate the bacterial load by applying the two methods is mainly due to the CFU method’s unreliability in counting bacteria aggregated, adherent, or in biofilm. As matter of fact, one CFU is constituted by several bacteria which, on the contrary, are really enumerated by the metabolic method of LCA. Therefore, if bacteria are aggregated, their number, obtained by CFU counts, is always lower than that obtained by the LCA. Of note, bacterial aggregation could be favored over time in the gel formulation. The LCA reagent preparation has been previously reported [19]. To perform the LCA, 1 g of the lipogel was immersed in LCA reagent (10 mL) to reach a concentration of 100 mg/mL. The lipogel in LCA reagent was covered with paraffin to create anaerobiosis at 37 °C. The initial red color changes to yellow due to metabolic reactions by *Lactobacillus* spp. This change was detected at 420 nm (yellow) every 30 min until 24 h. The time required for color change of LCA reagent from red to yellow was correlated to the initial microbial number by the correlation line reported by Giannini et al. [19]. Moreover, 100 μL of this sample, properly diluted, was plated on MRS agar plates to count CFUs.

### 5.5. Number of Bacteria Released in Physiological Solution or Simulated Saliva

An in vitro translational model was used to monitor the release of live bacteria, evaluating this parameter both in physiological solution (PS) and in simulated saliva (SS).

These two vehicles satisfactorily mimic a biological condition to profile the ability to release *lactobacillus* strains from the lipogel preparation. The gel was immersed in the liquid phase (PS or SS) prepared according to Marques et al. [27].

Briefly, at time 0, samples of weighted lipogel were immersed in LCA and tested to evaluate the total bacterial load at baseline. Subsequently, samples of lipogel were immersed in the PS or SS, and bacterial release was evaluated after 30 min and 2, 5, and 8 h. At each incubation time, 1 mL of solution was collected and mixed with 1 mL of LCA reagent to quantify the number of bacteria released. In parallel, to check the bacteria still present in the lipogel at each time, an aliquot of lipogel was collected and immersed in 1 mL of LCA reagent. The number of loaded or released bacteria was normalized at 1 g of lipogel.

The bacterial load of the lipogel was counted only with LCA. CFU count was unreliable in the enumeration of lactobacilli in the lipogel. Conversely, bacterial release in PS or SS was counted using both the LCA and CFU methods.

### 5.6. In Vitro Evaluation of Irritation on Human Oral Mucosa and TEWL Measurement

A three-dimensional model of in vitro reconstructed oral mucosa (HOE) to evaluate the tolerability and safety of the mucoadhesive gel was used.

This cell line (HOE) was derived from a human squamous cell carcinoma, taken from the buccal mucosa, and placed in a chemically defined medium. In culture, these cells form a highly differentiated multi-layered epithelium model, mimicking organized cell layers analogous to that present in human oral mucosa and lacking a stratum corneum. For this complexity, this model was chosen as first, before the gingival ones.

The use of HOE provided information on the possible irritative effect of the gel on the human oral mucosa.

Gel AL0020 was topically applied to the HOE model, to evaluate the tolerability of the sample, and the viability degree of the layer was observed after contact with the sample. These observations provided information about the capability of samples to change cell viability and retain fluids, ensuring a good level of barrier function to the mucous membrane.

The MTT test was used to measure cell viability considering the results above as a defined threshold as indicative of the non-irritating effect of the samples. The cut-off level set in this assay was 50% of the negative control.

Transepidermal water loss (TEWL) is a key indicator of skin barrier function, and the ability to measure it accurately is essential in a wide range of clinical and personal care applications. In this test, the measurement of TEWL was performed on the mucosal inserts to verify their greater or lesser surface permeability with a Vapometer (Delfin). This instrument measures transepidermal water loss as evaporation rate in g/m^2^h through a humidity sensor that is located inside a cylindrical measuring chamber. This measuring chamber is closed by the mucosal support during measurements and is therefore not affected by ambient air flows. The sensor monitors the increase in relative humidity (RH) inside the chamber during the measurement phase, and the evaporation rate value (g/m^2^h) is automatically calculated from the increase in RH.

### 5.7. In Vitro Evaluation of Irritative Potential, Protective Efficacy, and Soothing Effect on Gingival Epithelium

Another test used to evaluate the mucoadhesive gel was a three-dimensional model of in vitro reconstructed gingival epithelium (HE).

This model was created by culturing normal human gingival cells on an inert polycarbonate filter, at the air–liquid interface, in a chemically defined medium. In culture, these cells form a highly differentiated multi-layered epithelium pattern histologically similar to the outer cell layers of human gum.

Such a support is treated topically with the tested product in order to study its irritative potential, its protective efficacy, and its soothing properties.

For the irritative potential study, the product is applied directly on the inserts and the treatment is maintained for four hours. This test evaluates whether the application of the sample under analysis can decrease cell viability in the inserts, proving in this way an irritative activity.

For the protective study, inserts are treated topically with the sample under test with a concomitant aggressive irritant product at the same time (sodium dodecyl sulphate 0.5%). In this case, the possible decrease of cell viability and the possible increase of Interleukin-1 alfa (IL-1α) expression can be informative about a product’s protection properties.

The soothing test is performed exposing the inserts to a mild irritant treatment with lactic acid 1% for two hours, followed by the application of the product under analysis for four hours during the first day and for a further four hours during the next day. The presence of the sample in analysis, if it acts as a soothing factor, can facilitate the recovery of the damage caused by the initial irritant treatment and thus increase the cellular viability and decrease IL-1α expression of the inserts at the end of the study.

Cell viability is measured by considering the conversion by cellular enzymes of the yellow salt of MTT into a water-insoluble blue compound called “formazan”. The amount of formazan formed during a defined contact period is proportional to the number of living cells present in the analyzed culture. IL-1α expression is measured by ELISA test.

### 5.8. Clinical Evaluation, Assessment of the Microbiome, and Self-Assessment Questionnaire of a Cosmetic Product for Oral Use

For a clinical evaluation of the activity of Tau-Marin gel, 20 volunteers applied AL0020 to their gums every evening, once a day for one month. The purpose of the study was to evaluate the ability of the product to rebalance the gingival mucosa altered by erythema, erosions, leukoplastic lesions, and bleeding, before and after treatment. The treatment activity was evaluated by a dentist at the end of the 30 days.

Microbiome rebalancing activity of the product is supported by the following:

No alteration of the microbiota.

Rebalancing of the bacterial species present.

A clinical-dermatological evaluation of the condition of gums was performed to assess the presence of erythema, gum bleeding, erosions, and leukoplastic lesions before and after treatment.

All clinical assessments were scored according to the following 4-point scale:

0 = absent; 1 = mild; 2 = moderate; 3 = severe.

The effectiveness of the product is evidenced by the decrease at the end of the treatment for erythema and gum bleeding parameters, whereas it is supported by the maintenance of absence of erosions and leukoplastic lesions.

At the end of the test, the volunteers expressed their subjective opinion on the effectiveness and pleasantness of the treatment by filling out a self-evaluation questionnaire.

## Figures and Tables

**Table 1:** Name, source, International Nomenclature of Cosmetic Ingredients (INCI), and percentage of ingredients in the lipogel AL0038. MCT (Medium-Chain Triglycerides).

Commercial Name	Source	INCI	%
LABRAFAC^®^ caprylic (C8)/Capric (C10) MCT oil	Gattefossè	Caprylic/Capric Triglyceride	75.25
Ethylcellulose	Asha Cellulose	Ethylcellulose	5.00
COMPRITOL^®^ 888 CG	Gattefossè	Glyceryl Behenate	1.00
AEROSIL^®^ R 972	Evonik	Hydrophobic fumed silica/Silica dimethyl silylate	5.00
BENECEL™ K4M	Ashland	Hydroxypropyl Methylcellulose	8.50
BENECEL™ K100M	Ashland	Hydroxypropyl Methylcellulose	5.00
Mint Flavour	Farotti	-	0.25

**Table 2:** Bacterial strain, percentage and theorical title used in the preparation, and minimum titer of the bacterial consortium in the final preparation.

Bacterial Strain	%	Bacteria into the Tau-Marin Gel (Theorical Title)		AL0020 (Time = 0)
		in 100 g gel	in 1 g gel	
*L. rhamnosus* SP1	1	3.0 × 10^11^ UFC/g	3.0 × 10^9^ UFC/g	>4 × 10^9^ UFC/g
*L. helveticus* SP27	1	2.0 × 10^11^ UFC/g	2.0 × 10^9^ UFC/g	
*L. paracasei* CBA-L87	1	1.0 × 10^11^ UFC/g	1.0 × 10^9^ UFC/g	

**Table 3:** The final formulation of Tau-Marin mucoadhesive gel.

Commercial Name	Source	INCI	%
LABRAFAC^®^ caprylic (C8)/Capric (C10) MCT oil	Gattefossè	Caprylic/Capric Triglyceride	71.05
Ethylcellulose	Asha Cellulose	Ethylcellulose	5.00
COMPRITOL^®^ 888 CG	Gattefossè	Glyceryl Behenate	1.00
AEROSIL^®^ R 972	Evonik	Hydrophobic fumed silica/Silica dimethyl silylate	5.00
BENECEL™ K4M	Ashland	Hydroxypropyl Methylcellulose	8.50
BENECEL™ K100M	Ashland	Hydroxypropyl Methylcellulose	5.00
Aloe vera gel Podwer regular 200X	Terry Laboratories LLC	Aloe Barbadensis Leaf Extract	0.20
Blueberry (1:4) dry extract	LaBioTRE	Vaccinium Myrtillus Fruit Extract	0.40
Mallow leaves (1:4) dry extract	LaBioTRE	Malva Sylvestris Leaf Extract	0.60
Mint Flavour	Farotti	Flavour	0.25
*L. rhamnosus* SP1	CSL	Lactobacillus Ferment	1.00
*L. helveticus* SP27	CSL	Lactobacillus Ferment	1.00
*L. paracasei* CBA-L87	CSL	Lactobacillus Ferment	1.00

**Table 4:** Combination used in the competition test.

Items	Pathogen Strain (s)	Against Probiotic Strain (s)
1	Five single pathogens	*L. rhamnosus* SP1
2	Five single pathogens	*L. paracasei* CBA-L87
3	Five single pathogens	*L. helveticus* SP27
4	Five single pathogens	AL0038 (Neutral gel)
5	Five single pathogens	AL0039 (gel containing three probiotic strains)
6	Five single pathogens	AL0019 (gel containing three botanical extracts)
7	Five single pathogens	AL0020 (gel containing 3 probiotic strains + 3 extracts)
8	Five single pathogens	Aloe vera extract
9	Five single pathogens	Blueberry extract
10	Five single pathogens	Mallow extract
11	Five pathogenic bacteria (consortium)	*L. rhamnosus* SP1
12	Five pathogenic bacteria (consortium)	*L. paracasei* CBA-L87
13	Five pathogenic bacteria (consortium)	*L. helveticus* SP27
14	Five pathogenic bacteria (consortium)	AL0038 (Neutral gel)
15	Five pathogenic bacteria (consortium)	AL0039 (gel containing three probiotic strains)
16	Five pathogenic bacteria (consortium)	AL0019 (gel containing three botanical extracts)
17	Five pathogenic bacteria (consortium)	AL0020 (gel containing 3 probiotic strains + 3 extracts)
18	Five pathogenic bacteria (consortium)	Aloe vera extract
19	Five pathogenic bacteria (consortium)	Blueberry extract
20	Five pathogenic bacteria (consortium)	Mallow extract

**Table 5:** Assessment of the Antimicrobial Activity against “red complex” pathogenic strains. Results of agar diffusion well assay were obtained in duplicate.

Tested Conditions in Duplicate		Pathogen Blend	*P. melan*	*P. gingivalis*	*T. denticola*	*T. forsithia*	*A. actin*
			DSM7089	DSM20709	DSM14222	DSM102835	DSM8324
Uncutted well	CT−	/	/	/	/	/	/
Chlorexidine 0.2%	CT+	+++	+++	+++	+++	+++	+++
AL0038 gel	Mint flavor	/	/	/	/	+	/
AL0039 gel	Mint flavor + three probiotics	+	+	+	+	+	+
AL0019 gel	Mint flavor + three botanicals	+	+	++	+	+	+
**AL0020 gel**	complete formulation	**++**	**+**	**++**	**+**	**++**	**++**
*L. rhamnosus* SP1	DSM21690	/	/	+	/	/	/
*L. helveticus* SP27	DSM29575	/	/	/	/	/	+
*L. paracasei* CBA-L87	LMG-26420)	/	+	+	/	+	/
Aloe vera	Gel powder regular 200X	+	+	+	/	+	+
Blueberries (1:4)	Dry extract	+	+	/	/	/	/
Mallow leaves (1:4)	Dry extract	+	+	+	/	+	+

“+” indicates a weak inhibition; “++” indicates intermediate inhibition/colonies rarefaction; “+++” indicates strong inhibition/clear halo. “/” indicates no inhibition.

**Table 6:** Number of lactobacilli contained in the lipogel AL0020 and released in physiological solution (PS) through Lacto-Counter Assay (LCA) and Colony Forming Unit (CFU).

Time (Months)	0 Min	30 Min			2 h			5 h			8 h		
	1 g (LCA)	1 g (LCA)	1 mL PS (LCA)	1 mL PS (CFU)	1 g (LCA)	1 mL PS (LCA)	1 mL PS (CFU)	1 g (LCA)	1 mL PS (LCA)	1 mL PS (CFU)	1 g (LCA)	1 mL PS (LCA)	1 mL PS (CFU)
T0	5.1 × 10^9^	3.9 × 10^9^	8.6 × 10^6^	6.7 × 10^6^	2.5 × 10^9^	2.0 × 10^8^	8.0 × 10^7^	1.3 × 10^9^	8.7 × 10^8^	1.3 × 10^8^	1.6 × 10^8^	2.3 × 10^9^	5.2 × 10^8^
T1	5.0 × 10^9^	4.3 × 10^9^	8.3 × 10^6^	6.3 × 10^6^	2.6 × 10^9^	1.6 × 10^8^	7.5 × 10^7^	1.5 × 10^9^	8.9 × 10^8^	1.8 × 10^8^	1.3 × 10^8^	2.8 × 10^9^	6.0 × 10^8^
T3	5.0 × 10^9^	4.5 × 10^9^	9.1 × 10^6^	8.4 × 10^6^	2.2 × 10^9^	2.5 × 10^8^	9.7 × 10^7^	1.2 × 10^9^	1.2 × 10^9^	2.3 × 10^8^	1.5 × 10^8^	3.0 × 10^9^	6.4 × 10^8^
T6	4.9 × 10^9^	4.0 × 10^9^	3.1 × 10^7^	9.5 × 10^6^	1.8 × 10^9^	4.5 × 10^8^	1.7 × 10^8^	9.2 × 10^8^	2.0 × 10^9^	4.8 × 10^8^	1.0 × 10^8^	3.3 × 10^9^	8.9 × 10^8^
T9	4.8 × 10^9^	4.0 × 10^9^	4.4 × 10^7^	9.1 × 10^6^	1.2 × 10^9^	6.3 × 10^8^	1.2 × 10^8^	9.0 × 10^8^	2.2 × 10^9^	4.9 × 10^8^	1.1 × 10^8^	3.6 × 10^9^	8.4 × 10^8^
T12	4.8 × 10^9^	3.9 × 10^9^	4.7 × 10^7^	1.0 × 10^7^	1.3 × 10^9^	7.3 × 10^8^	1.6 × 10^8^	8.6 × 10^8^	2.4 × 10^9^	5.6 × 10^8^	1.0 × 10^8^	3.9 × 10^9^	8.4 × 10^8^

Time 0 min refers to the number of lactobacilli in the lipogels, whereas timepoints 30 min and 2, 5, and 8 h indicate the number of bacteria remaining in the lipogel (1 g) or released (1 g/mL) in PS after different incubation times. The number of bacteria loaded and released from lipogels to PS was measured at the moment of the preparation (T0) and after 1 (T1), 3 (T3), 6 (T6), 9 (T9), and 12 (T12) months. The standard deviation (SD), ranging between 0.1 and 0.3 for all the means, was not reported.

**Table 7:** Number of lactobacilli contained in the lipogel AL0020 and released in simulated saliva (SS) through Lacto-Counter Assay (LCA) and Colony Forming Unit (CFU).

Time (Months)	0 Min	30 Min			2 h			5 h			8 h		
	1 g (LCA)	1 g (LCA)	1 mL SS (LCA)	1 mL SS (CFU)	1 g (LCA)	1 mL SS (LCA)	1 mL SS (CFU)	1 g (LCA)	1 mL SS (LCA)	1 mL SS (CFU)	1 g (LCA)	1 mL SS (LCA)	1 mL SS (CFU)
T0	5.1 × 10^9^	4.1 × 10^9^	9.7 × 10^6^	3.9 × 10^6^	2.8 × 10^9^	1.3 × 10^8^	6.3 × 10^7^	1.6 × 10^9^	7.3 × 10^8^	2.5 × 10^8^	1.8 × 10^8^	2.6 × 10^9^	6.1 × 10^8^
T1	5.0 × 10^9^	4.1 × 10^9^	9.4 × 10^6^	4.4 × 10^6^	2.2 × 10^9^	1.8 × 10^8^	5.9 × 10^7^	1.3 × 10^9^	8.0 × 10^8^	2.0 × 10^8^	1.6 × 10^8^	2.8 × 10^9^	6.5 × 10^8^
T3	5.0 × 10^9^	4.3 × 10^9^	1.1 × 10^7^	5.9 × 10^6^	2.5 × 10^9^	2.8 × 10^8^	7.4 × 10^7^	1.3 × 10^9^	9.7 × 10^8^	3.1 × 10^8^	1.3 × 10^8^	3.0 × 10^9^	7.5 × 10^8^
T6	4.9 × 10^9^	4.0 × 10^9^	3.4 × 10^7^	8.9 × 10^6^	2.2 × 10^9^	3.8 × 10^8^	9.4 × 10^7^	9.3 × 10^8^	1.7 × 10^9^	3.8 × 10^8^	1.0 × 10^8^	3.5 × 10^9^	8.6 × 10^8^
T9	4.8 × 10^9^	4.1 × 10^9^	3.1 × 10^7^	7.9 × 10^6^	1.9 × 10^9^	4.3 × 10^8^	1.0 × 10^8^	9.2 × 10^8^	2.3 × 10^9^	4.0 × 10^8^	9.8 × 10^7^	3.7 × 10^9^	7.9 × 10^8^
T12	4.8 × 10^9^	3.8 × 10^9^	4.2 × 10^7^	1.1 × 10^7^	1.5 × 10^9^	7.3 × 10^8^	1.3 × 10^8^	8.5 × 10^8^	2.7 × 10^9^	4.7 × 10^8^	9.0 × 10^7^	4.0 × 10^9^	8.3 × 10^8^

Time 0 min refers to the number of lactobacilli in the lipogels, whereas timepoints 30 min and 2, 5, and 8 h indicate the number of bacteria remaining in the lipogel (1 g) or released (1 g/mL) in SS after different incubation times. The number of bacteria loaded and released from lipogels to SS was measured at the moment of the preparation (T0) and after 1 (T1), 3 (T3), 6 (T6), 9 (T9), and 12 (T12) months. The standard deviation (SD), ranging between 0.1 and 0.3 for all the means, was not reported.

**Table 8:** Cellular viability of Tau-Marin Gel, checked after 24 h, using MTT titration and analyzed for IL-1α expression.

	MTT Test—Cellular Viability (±S.D.)	
Negative control (physiological solution)	100.0%	(±6.6)
Positive control (SDS 0.5%)	1.9%	(±0.1)
Tau-Marin gel AL0020	113.9%	(±8.5)

**Table 9:** Protective effect against an irritant agent (0.5% SDS) by pre-treating inserts with Tau-Marin gel.

	MTT Test—Cellular Viability (±S.D.)	
Negative control (physiological solution)	100.0%	(±6.6)
Positive control (SDS 0.5%) for 1 h	1.9%	(±0.1)
Tau-Marin gel AL0020 + irritative agents (SDS 0.5%) for 1 h	108.0%	(±10.9)

**Table 10:** The TEWL values of the barrier function of the mucous membrane, both at the end of the four hours of application and after the post-treatment rest period.

Treatment	% Cellular Viability (±S.D.)	TEWL (g/m^2^h)		
		Before Treatment	After Treatment	After Rest
Negative control	100.0 (±2.7)	12.9 (±0.8)	16.8 (±0.7)	18.8 (±1.4)
Positive control	2.9 (±1.7)	13.4 (±0.7)	19.3 (±0.7)	17.6 (±1.5)
Tau-Marin gel	87.6 (±1.7)	14.9 (±0.5)	12.6 (±1.1)	14.4 (±2.7)

**Table 11:** Relative frequencies of the answers given to the questions in the final questionnaire.

Question		Opinion Expressed			
		Excellent (4)	Good (3)	Sufficient (2)	Insufficient (1)
A	How do you evaluate product’s applicability?	32%	42%	16%	11%
B	How do you judge product’s adhesiveness?	37%	37%	21%	5%
C	How do you judge product’s texture?	32%	26%	32%	1%
D	How do you judge the taste of the product?	26%	21%	53%	-
E	How do you judge the persistence of the flavour?	26%	26%	37%	11%
F	How do you judge the pleasantness of night use?	16%	26%	47%	11%
G	How do you rate the ease of removing the product in the morning?	68%	21%	11%	-
H	How do you evaluate the product’s ability to relieve gum inflammation?	53%	47%	-	-
I	How do you evaluate the product’s ability to relieve gum irritation?	58%	42%	-	-
L	How do you evaluate the product’s ability to calm gingivitis?	47%	53%	-	-
M	How do you evaluate the product’s ability to calm gum bleeding?	53%	42%	5%	-
N	How do you evaluate the product’s ability to mitigate the annoyance of heat/cold?	37%	58%	5%	-
O	Did you feel a tingling, reddening sensation or any other alteration in the sensitivity of gum mucosae while using the product?	100%	-	-	-
P	Would you buy the product again?	26%	37%	26%	11%
Q	How do you rate the product overall?	21%	63%	16%	-

**Table 12:** (Erythema) Comparison of initial data (T0) with those after 30 days of treatment with the product (TF).

T0	TF = 30 Days	Variation T0-TF	T0 vs. TF (*p*-Value)
Mean 1.6 (±SD 0.5)	Mean 0.7 (±SD 0.5)	−0.8	*p* < 0.0001

**Table 13:** (Gum Bleeding) Comparison of initial data (T0) with those after 30 days of treatment with the product (TF).

T0	TF = 30 Days	Variation T0-TF	T0 vs. TF (*p*-Value)
Mean 1.5 (±SD 0.9)	Mean 0.3 (±SD 0.4)	−1.2	*p* < 0.0003

**Table 14:** (Erosion). Comparison of initial data (T0) with those after 30 days of treatment with the product (TF).

T0	TF = 30 Days	T0 vs. TF (*p*-Value)
Mean 0.1 (±SD 0.3)	Mean 0.0 (±SD 0.0)	*p* < 0.35

**Table 15:** (Leukoplastic lesions). Comparison of initial data (T0) with those after 30 days of treatment with the product (TF).

T0	TF = 30 Days	T0 vs. TF (*p*-Value)
Mean 0.2 (±SD 0.5)	Mean 0.0 (±SD 0.0)	*p* < 0.37

**Table 16:** The components (gel formulations and single ingredients) investigated in the competition test.

Items	Composition	Batch
Uncutted well	Negative Control (CT−)	-
Chlorexidine 0.2%	Positive Control (CT+)	-
AL0038	Gel-mint flavour (Neutral Gel: mucoadhesive medium only)	IR0321
AL0039	Gel-mint flavour + 3 probiotics	IR0322
AL0019	Gel-mint flavour + 3 botanical extracts	IR0323
AL0020	Gel-mint flavour + 3 botanical extracts + 3 probiotics	L1538 K
*L. rhamnosus* SP1	DSM 21690	22000690
*L. helveticus* SP27	DSM 29575	22003367
*L. paracasei* CBA-L87	LMG-26420	22004203
Aloe vera	gel Powder regular 200X	19007530
Blueberries	(1:4) dry extract	22003192
Mallow leaves	(1:4) dry extract	22003567

## Data Availability

All experimental data associated with this study have been included and detailed in this manuscript and in the S.I.. There are no further data available. Original reports are available from the corresponding author upon request. Results of this research are protected by an international patent application, No. WO2022/161893A1, titled: “Buccal mucoadhesive gel containing probiotics for use in the prevention and treatment of periodontal diseases”.

## Supplementary Materials

The following supporting information can be downloaded at: https://www.mdpi.com/article/10.3390/gels9080607/s1, Figure S1: (A) Alpha diversity index distributions. (B): Alpha diversity pairwise differences. (C): Beta diversity pairwise differences; Figure S2: Grouped bar plots; Figure S3: TEWL results of Tau-Marin gel versus negative and positive control.

## Abbreviations

AL	(Alfasigma)
CFUs	(Colony Forming Units)
HE	(Haematoxylin-Eosin-staining)
HOE	(Human Oral Epithelium)
HPMC	(Hydroxy-Propyl-Methylcellulose)
INCI	(International Nomenclature of Cosmetic Ingredients)
LCA	(Lacto-Counter Assay)
MCT	(Medium-Chain Triglycerides)
MDR	(Maximum Recovery Diluent)
MPs	(Modified Silicas)
MRS	(de Man, Rogosa and Sharpe) agar
MTT	(Colorimetric assay based on the enzymatic reduction of tetrazolium dye, which is chemically 3-(4,5-dimethylthiazol-2-yl)-2,5-diphenyltetrazolium bromide, for assessing cell metabolic activity)
PACs	(ProAnthoCyanidins)
PBS	(Phosphate Buffered Saline)
PS	(Physiological Solution)
SDS	(Sodium Dodecyl Sulphate)
SS	(Simulated Saliva)
TEWL	(TransEpidermal Water Loss)
