# Supplementary material for: Tau-Marin Mucoadhesive Gel for Prevention and Treatment of Gum Diseases
Source: Gels. 2023 Jul 27;9(8):607. doi: 10.3390/gels9080607 (PMC10453578; doi:10.3390/gels9080607)
Supplement: Supplementary file 1 [file gels-09-00607-s001.zip › gels-2520737-supplementary.pdf]

Article

Tau-Marin Mucoadhesive Gel for Prevention and Treatment of Gum Diseases

Giuseppe Giannini [1,*], Irene Ragusa [2], Giulia Nerina Nardone [2], Sara Soldi [3], Marina Elli [3], Piera Valenti [4] and Luigi Rosa [4]

1 R&D Alfasigma S.p.A., Via Pontina, Km 30.400, 00071 Pomezia, Italy
2 Labomar S.p.a., Via F. Filzi, 33, 31036 Treviso, Italy; irene.ragusa@labomar.com (I.R.); giulianerina.nardone@labomar.com (G.N.N.)
3 AAT—Advanced Analytical Technologies Srl, Via P. Majavacca 12, 29017 Fiorenzuola d'Arda, Italy; sara.soldi@aat-taa.eu (S.S.); marina.elli@aat-taa.eu (M.E.)
4 Department of Public Health and Infectious Diseases, University of Rome La Sapienza, Piazzale A. Moro 5, 00185 Rome, Italy; piera.valenti@uniroma1.it (P.V.); luigi.rosa@uniroma1.it (L.R.)
* Correspondence: giuseppe.giannini@alfasigma.com

Supporting Information

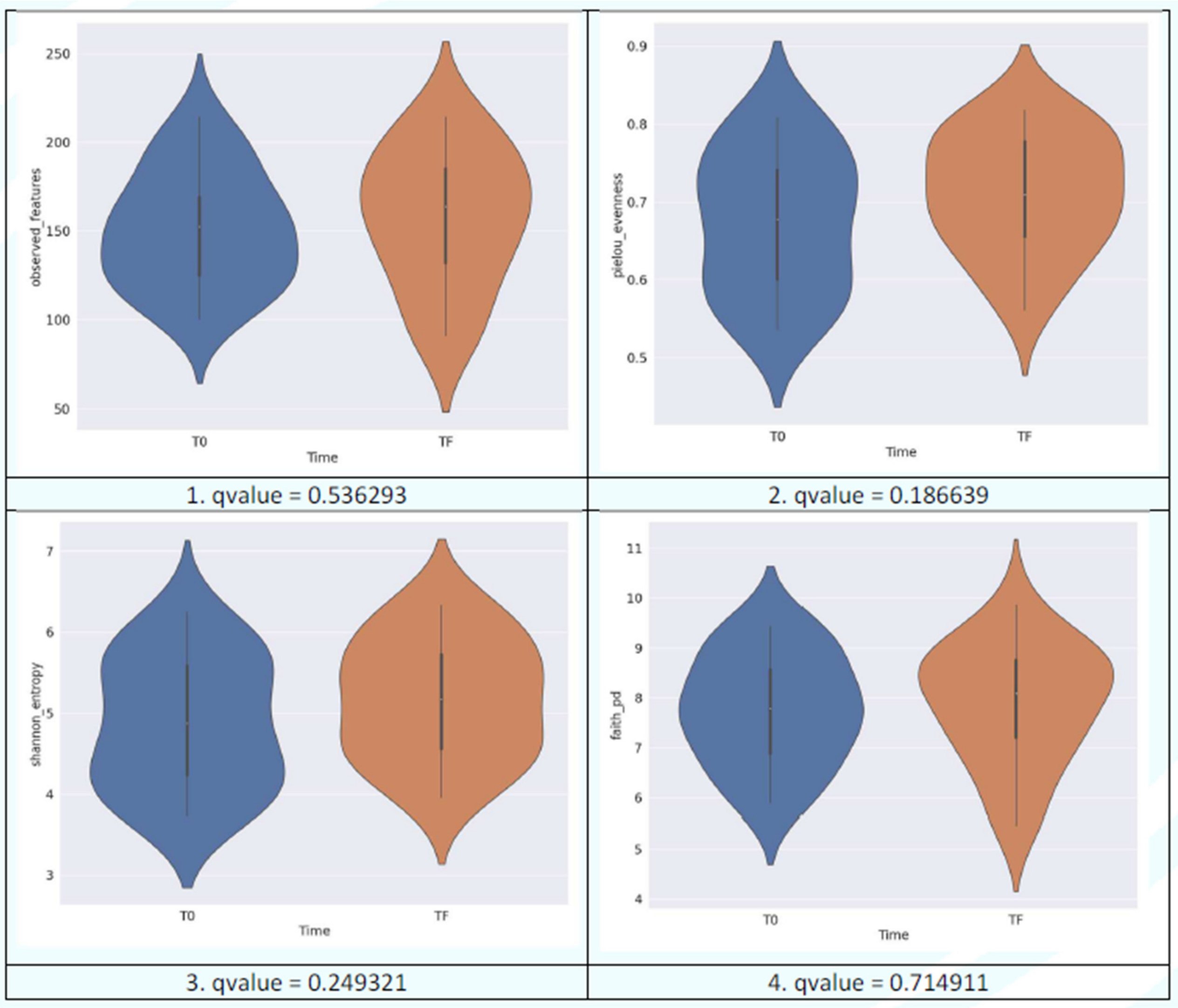

(**A**)

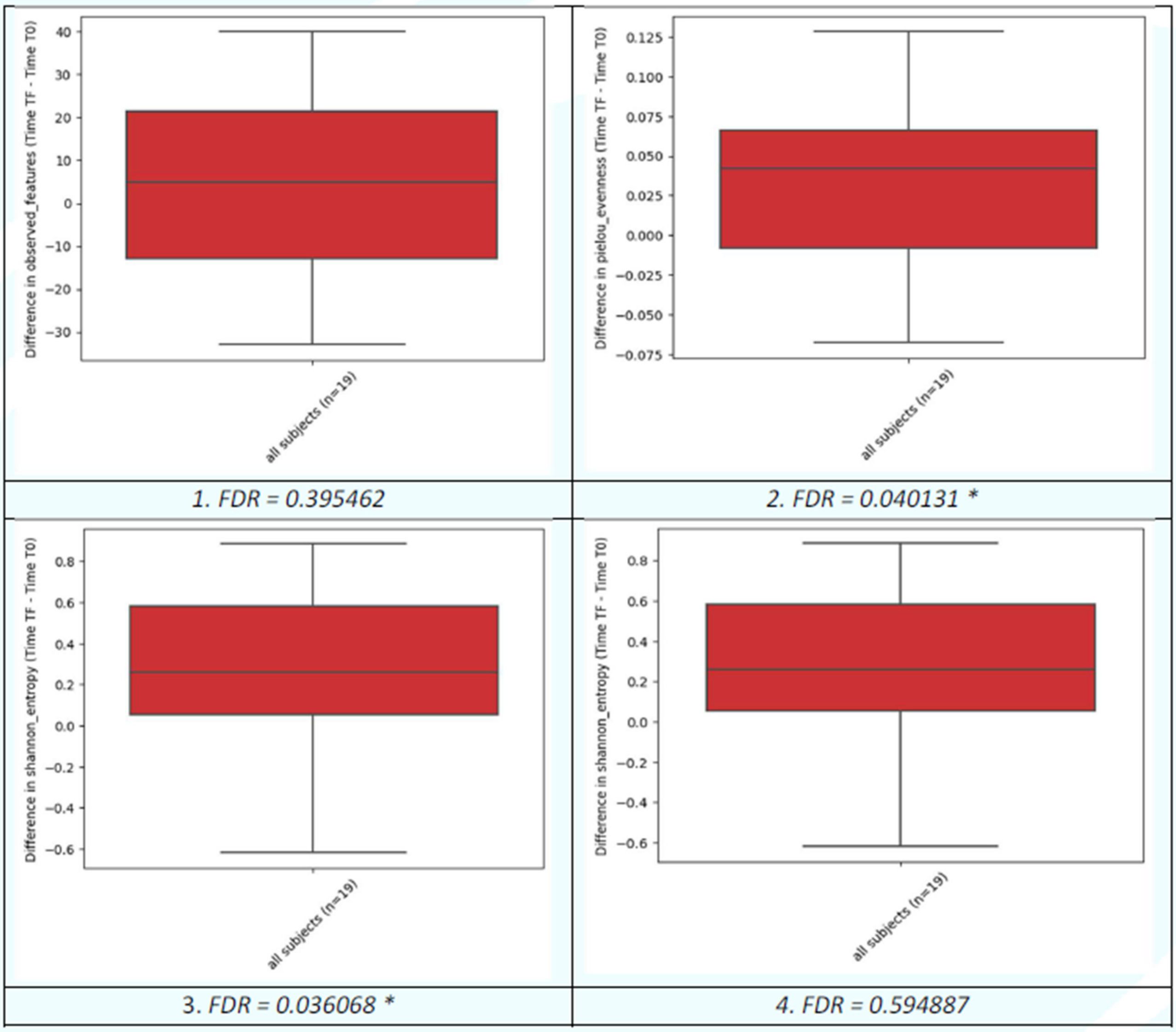
Difference in observed_features (Time TF - Time T0)
all subjects (n=19)
1. FDR = 0.395462
Difference in pielou_evenness (Time TF - Time T0)
all subjects (n=19)
2. FDR = 0.040131 *
Difference in shannon_entropy (Time TF - Time T0)
all subjects (n=19)
3. FDR = 0.036068 *
Difference in shannon_entropy (Time TF - Time T0)
all subjects (n=19)
4. FDR = 0.594887

(**B**)

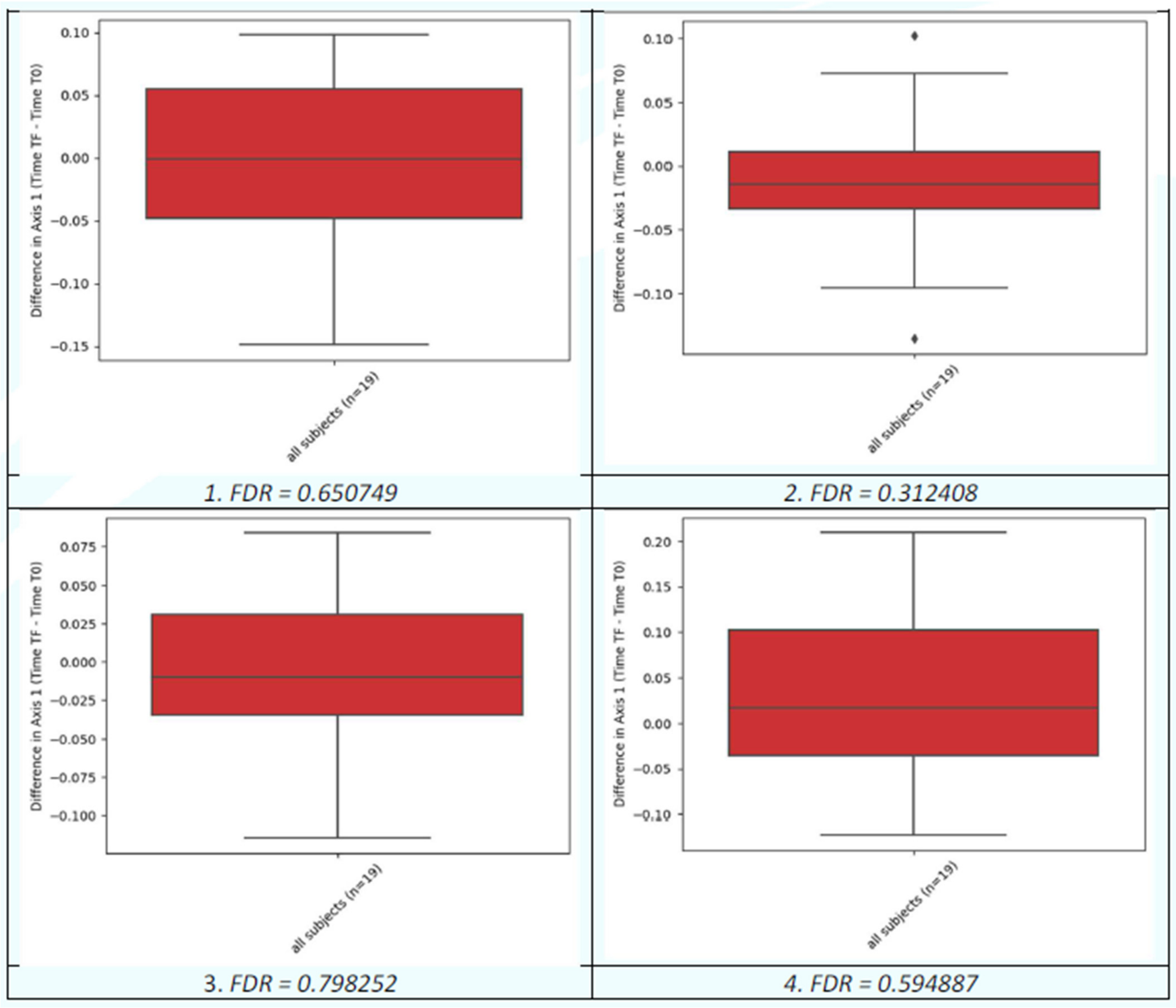

(**C**)

Figure S1: (**A**): Alpha diversity index distributions. Each violin plot shows a specific alpha diversity index distribution at the two time points (20 at T0 in blue, and 19 at TF in orange). The q-value of Kruskall Wallis test is indicated below the graph. (**1**) Observed features; (**2**) Pielou's evenness; (**3**) Shannon entropy; (**4**) Faith Phylogenetic Diversity. (**B**): Alpha diversity pairwise differences. Each boxplot shows the distribution of the differences of an alpha-diversity index between T0-TF within the same sample (pairwise difference). The FDR p-value of the Wilcoxon signed-rank test, used to determine if the mean of differences ranges from 0, is indicated below the graph. Sample S4 is excluded from the analysis, as not paired. (**1**) Observed features; (**2**) Pielou's evenness; (**3**) Shannon entropy; (**4**) Faith Phylogenetic Diversity. (**C**): Beta diversity pairwise differences. Each boxplot shows the distribution of the differences in the PCoA Axis1 of the beta-diversity metric between T0-TF within the same sample (pairwise difference). The FDR p-value of the Wilcoxon signed-rank test, used to determine if the mean of differences ranges from 0, is indicated below the graph. Sample S4 is excluded from the analysis, as not paired. (**1**) Bray Curtis; (**2**) Jaccard; (**3**) Unweighted Unifrac; (**4**) Weighted Unifrac.

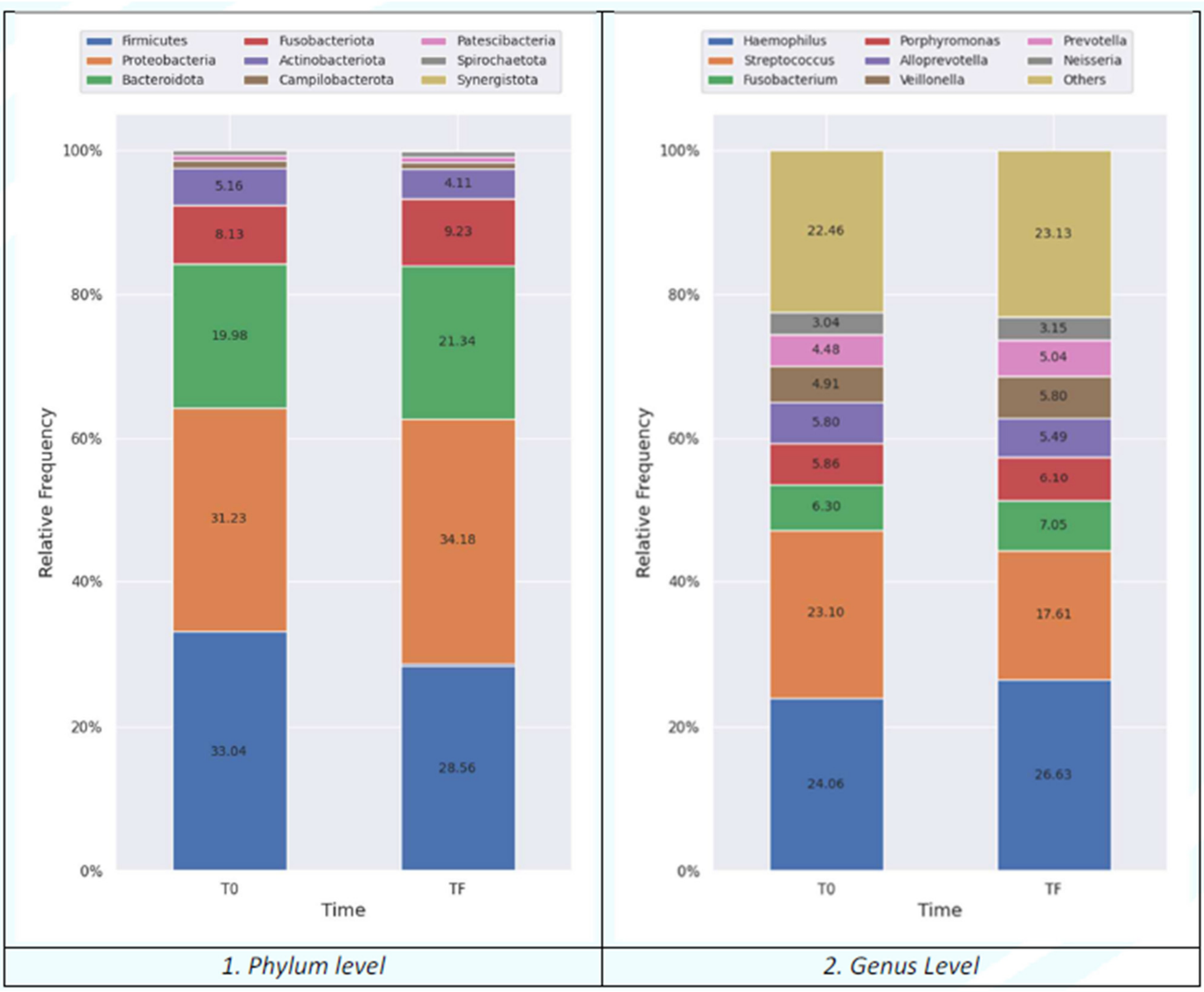

Figure S2. Grouped bar plots. Bar plots grouped on Time showing the relative abundance of the most abundant taxa at phylum (**1**) and genus (**2**) level. Relative abundance of each taxon is also shown.

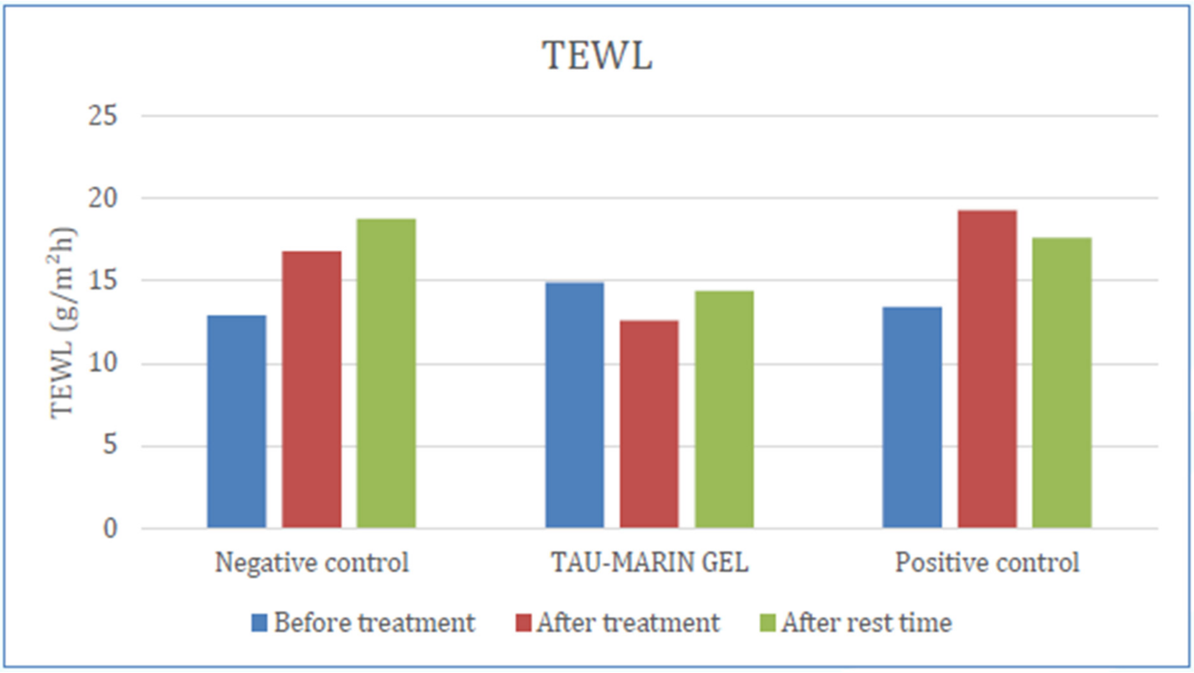

Figure S3. TEWL results of Tau-Marin gel versus negative and positive control, evaluated on the mucosal model.
